# A New Low‐Rate Stable Hydrogel Cathode for Aqueous Zn‐ion Batteries

**DOI:** 10.1002/cssc.202501942

**Published:** 2025-10-23

**Authors:** Roya Rajabi, Shichen Sun, Jamil Khan, Morgan Stefik, Kevin Huang

**Affiliations:** ^1^ Department of Mechanical Engineering University of South Carolina Columbia SC29201 United States of America; ^2^ Department of Chemistry and Biochemistry University of South Carolina Columbia SC29201 United States of America

**Keywords:** electrochemical active sites, hydrogel, low cycle rate, stability, Zn‐ion batteries

## Abstract

Aqueous Zn‐ion batteries (ZIBs) are attractive candidates for large‐scale energy storage owing to the abundance, low cost, and intrinsic safety of Zn metal. However, their practical application is hindered by poor cycle stability, especially at low current densities, due to cathode dissolution and limited electrochemically active sites (EAS). Herein, a hydrogel‐based cathode comprising ammonium vanadate, carbon black, and a Zn‐ion‐conducting carboxymethyl chitosan–acrylamide hydrogel matrix doped with Zn(ClO_4_)_2_ is reported. This design establishes a continuous Zn‐ion‐conducting network, thereby maximizing EAS density throughout the electrode volume. The ZIB with the hydrogel cathode exhibits outstanding cycling stability, with 77% capacity retention after 2000 cycles at 1 A g^−1^ and 75% retention after 1400 cycles at 0.5 A g^−1^, far surpassing conventional polyvinylidene fluoride‐based cathodes. In addition to retaining high EAS density, the hydrogel matrix also suppresses active material dissolution. These results demonstrate a new strategy for stabilizing ZIB cathodes and advancing long‐duration energy storage.

## Graphical abstract

A mixed ion‐ and electron‐conducting hydrogel enables low‐rate stability of aqueous Zn‐ion batteries.

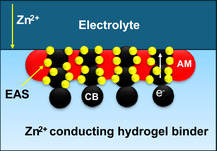

## 1 Introduction

The growing demand for safe, low‐cost, and sustainable energy storage technologies has intensified interest in aqueous Zn‐ion batteries (ZIBs). Zn anodes offer key advantages including a low redox potential (−0.76 V vs. SHE), high theoretical capacity (820 mAh g^−1^, 5,855 mAh cm^−3^), natural abundance, and intrinsic safety.^[^ ^1^, ^2^, ^3^ ^]^ Despite these merits, ZIB deployment is hampered by capacity fade and limited cycling stability. The primary challenges stem from hydrogen evolution reactions at the Zn anode and dissolution of vanadium‐ and manganese‐based cathodes into aqueous electrolytes, particularly under long‐duration, low‐rate cycling, where accumulation of alkaline byproducts and dissolution become more pronounced.^[^ ^4^, ^5^, ^6^, ^7^, ^8^, ^9^, ^10^, ^11^ ^]^

Extensive efforts in recent research have focused on cathode innovations to overcome these limitations. Mn‐based oxides offer high capacity and low cost but suffer from sluggish Zn^2^^+^ diffusion and dissolution; surface engineering with carbon nanotubes (CNT) and C_3_N_4_ has been demonstrated to help improve conductivity and stability.^[^ ^12^ ^]^ Vanadium‐based cathodes provide high capacity and multivalent redox activity yet face interlayer repulsion and dissolution issues.^[^ ^13^ ^]^ Recent strategies such as defect engineering in ammonium vanadates and halogen doping (e.g., iodine in sodium vanadates) have shown significant improvements in Zn^2^^+^ diffusion, lattice stability, and long‐term cycling performance.^[^ ^14^ ^]^

Conventional cathodes, typically comprising active material, carbon black (CB), polyvinylidene fluoride (PVDF) binder, and N‐methyl‐2‐pyrrolidone (NMP) solvent, suffer from discontinuous Zn‐ion transport pathways due to PVDF's hydrophobicity. Consequently, electrochemically active sites (EAS) are confined to the electrode–electrolyte interface, leading to underutilized capacity and accelerated degradation.^[^ ^15^, ^16^, ^17^, ^18^ ^]^ Water‐soluble binders such as carboxymethyl cellulose, hydroxyethyl cellulose, polyvinyl alcohol, and calcium alginate have been explored to improve interfacial ion transport.^[^ ^19^, ^20^, ^21^, ^22^, ^23^, ^24^ ^]^ To avoid binder‐induced transport limitations, Huang et al. developed a binder‐free MnO_2_/rGO cathode with improved flexibility and rate capability,^[^ ^25^ ^]^ and Yin et al. reported a freestanding V_2_O_5_/CNT paper electrode.^[^ ^26^ ^]^ To increase the conductivity of the cathode, Ma et al. used a conductive PEDOT binder combined with a 3D current collector to stabilize VO_2_ electrodes.^[^ ^27^ ^]^ More recently, Jaikrajang et al. and Dong et al. highlighted the impact of functional binder groups and interface engineering in improving cathode stability,^[^ ^19^, ^28^ ^]^ while Lu et al. optimized MnO_2_ binder‐electrolyte interfaces using hydroxyethyl cellulose.^[^ ^18^ ^]^

While these early studies clearly show that eliminating PVDF can enhance ZIB performance, none of them introduced Zn^2+^‐conducting binder into cathodes. This approach is fundamentally different in that the Zn^2+^‐conducting binder not only replaces PVDF but also creates a continuous ion‐conducting and electrochemically active network throughout the bulk of electrode. This unique design couples ionic conduction with water activity reduction directly within the binder matrix, which is absent in earlier PVDF‐free strategies.

The Zn^2+^‐conducting binder we are presenting here is based on a hydrogel made of carboxymethyl chitosan acrylamide (CMCS–AM) doped with Zn(ClO_4_)_2_. This hydrogel matrix embeds hydrated ammonium vanadate (NVOH) active material and CB, creating a continuous Zn‐ion‐conducting network with high EAS density throughout the electrode volume. To prove the new Zn^2+^ conducting hydrogel cathode concept, we evaluate its electrochemical performance in a full battery cell and compare it side‐by‐side with PVDF‐based controls. Additional pre‐ and post‐cycling characterizations on hydrogel cathode are conducted to facilitate the understanding of the improved performance.

## 2 Experimental Section

### 2.1 Materials

Zinc perchlorate hexahydrate (Zn(ClO_4_)_2_·6H_2_O, 98%), CMCS, AM, *N*,*N*′‐methylenebisacrylamide (MBAA), ammonium persulfate (APS), and Zn foil (>99.9%, 250 μm thickness) were purchased from Sigma‐Aldrich and used as received.

NVOH was synthesized from ammonium metavanadate (NH_4_VO_3_, Sigma‐Aldrich) following a reported method.^[^ ^29^ ^]^ Briefly, 0.585 g NH_4_VO_3_ was dissolved in 30 mL deionized water under stirring for 30 min. The pH was then adjusted to 1.3 using 1 m H_2_SO_4_, followed by an additional 30 min of stirring. The resulting dark red solution was transferred into a Teflon‐lined stainless‐steel autoclave and heated at 100 °C for 48 h. The red precipitate obtained was washed thoroughly with deionized water and ethanol and dried at 80 °C for 12 h. The phase purity of the product was confirmed by X‐ray diffraction (XRD), (see Figure S1, Supporting Information).

### 2.2 Preparation of Conventional PVDF Cathode

Conventional cathodes were fabricated by preparing a slurry of NVOH, CB, and PVDF in a weight ratio of 60:26:14, dispersed in N‐methyl‐2‐pyrrolidone (NMP). The slurry was cast onto Ti foil using a doctor blade and dried at 80 °C overnight. The effective NVOH loading was ≈0.5 mg cm^−2^.

### 2.3 Preparation of New Hydrogel Cathode

**Figure 1:**
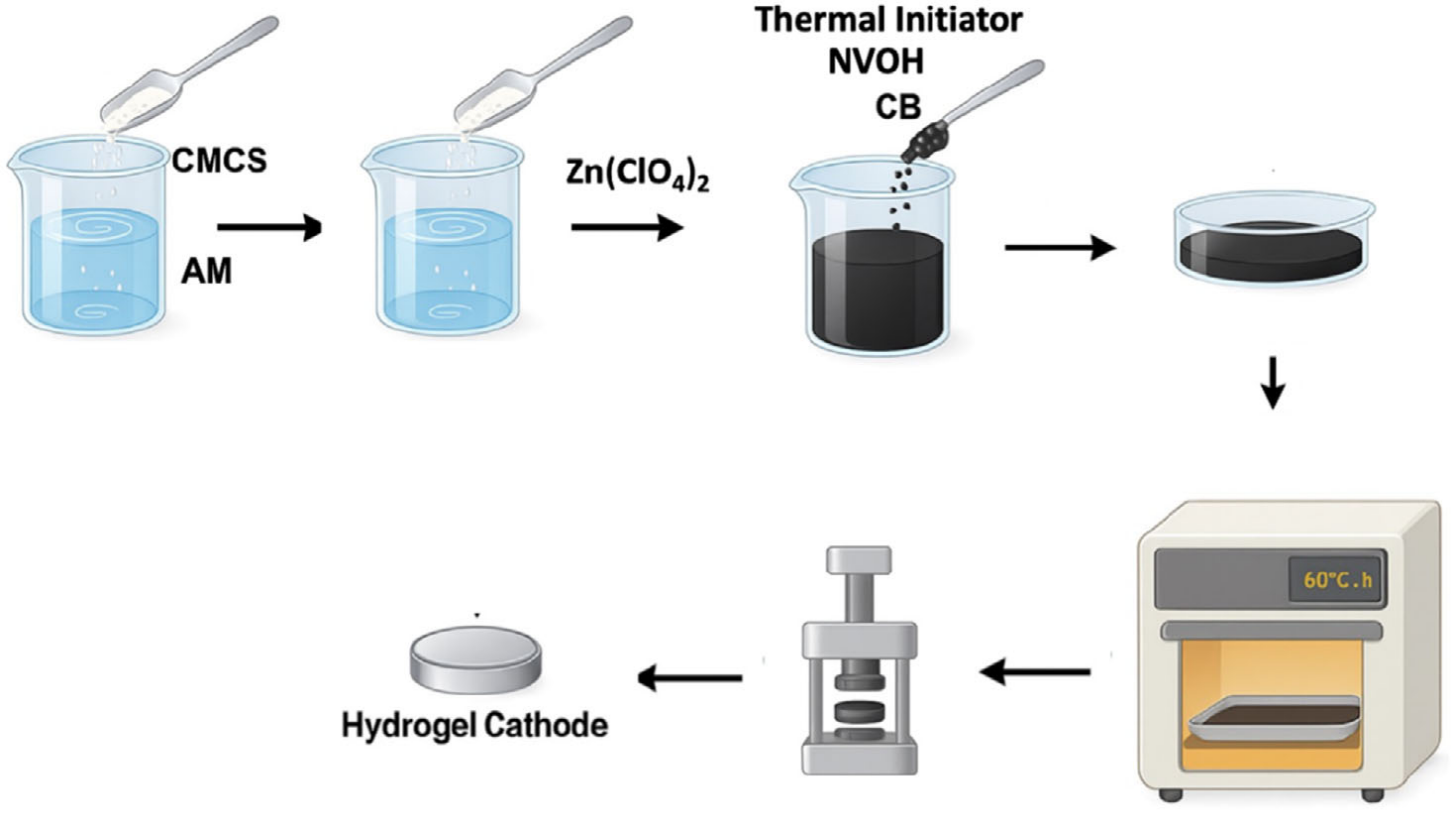
Schematic illustration of hydrogel‐based cathode synthesis.

Hydrogel cathodes were prepared by incorporating NVOH and CB into a CMCS–AM hydrogel matrix. In a typical batch, 25 mg CMCS was dissolved in 5 mL deionized water, followed by addition of 1.5 g AM monomer and 3.6 g Zn(ClO_4_)_2_. After homogenization, 150 mg NVOH and 60 mg CB were dispersed into 4 mL of the precursor solution. Polymerization was initiated by adding 20 mg APS and 1 mg MBAA, and the mixture was cured at 60 °C for 2 h. The resulting composite hydrogel thin film comprised a Zn‐ion‐conducting hydrogel matrix, NVOH, and CB. Film thickness varied depending on mold dimensions; for this study, the average thickness was ≈270 μm, with an effective NVOH loading of 2.3 mg cm^−2^. The overall synthesis process is illustrated in **Figure** **1**.

### 2.4 Electrochemical Characterization

The standard CR2032 coin cells were used to characterize the performance of the hydrogel cathode. To do so, the cathode hydrogel thin film was first punched into the desired size and laminated in sequence with the electrolyte separator and Zn anode within a CR2032 case. A Zn foil (200 μm thick) was used as the anode, 2 m Zn(ClO_4_)_2_ with glass fiber separator (20 μm thick, Whatman, GF/A) as the electrolyte and either hydrogel or PVDF‐based cathodes. Galvanostatic charge/discharge (GCD) cycling was conducted between 0.25 and 1.7 V at current densities of 0.3, 0.5, 0.6, 1.0, 2.0, and 4.0 A g^−1^. Specific capacities were calculated based on the mass of active material.

Electrochemical impedance spectroscopy (EIS) was employed to determine the ionic conductivity of the synthesized hydrogel and to compare the electrode polarization resistances of the full battery cell before and after cycling. All EIS measurements were carried out under the open‐circuit conditions within a frequency range of 1–10^5^ Hz with an AC stimulus of 10 mV.

### 2.5 Hydrogel Characterization

Fourier transform infrared (FTIR) spectroscopy (Agilent Cary 630) was employed to characterize hydrogel functional groups. XRD (Rigaku D/MAX‐2100, Cu Kα, *λ* =  1.54056 Å, 40 kV, 30 mA) was used to confirm the phase composition of NVOH, collected over a 2*θ* range of 10°–80° with a step resolution of 0.01°. Water activity (*a*_w_) of the hydrogel was measured at room temperature using a commercial water activity meter (Aqualab 4TE).

For EIS‐based ionic conductivity measurement of the hydrogel, membrane samples with dimension of *ϕ*14 mm, 270 μm thickness were sandwiched between two stainless‐steel electrodes. Ionic conductivity (*σ*) was calculated from ohmic resistance (*R*) using Ohm's law with the known sample thickness (*d*) and surface area (*A*). The temperature‐dependence of the ionic conductivity was also measured within the temperature range of 22–60 °C, from which the activation energy (*E*_a_) of the ionic conduction was determined using the Arrhenius equation.

## 3 Results and Discussion

### 3.1 Bulk Properties of the CMCS‐Based Hydrogel

**Figure 2:**
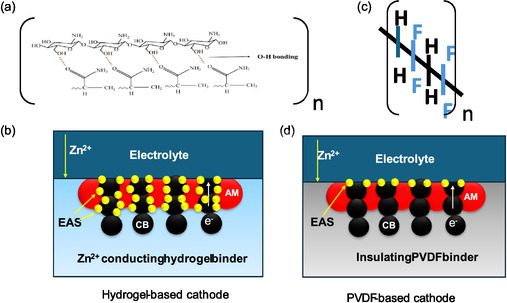
a) Molecular structure of CMCS‐based hydrogel; b) EAS region in hydrogel cathodes; c) molecular structure of PVDF; and d) EAS region in PVDF cathodes.

The unique role of the Zn^2+^‐conducting hydrogel binder in enabling efficient charge transport is schematically compared with the conventional PVDF binder in **Figure** **2**. In the hydrogel cathode, Figure 2a, the interconnected polymer network, enriched with –NH_2_, –COOH, and –OH groups, not only anchors water molecules but also establishes continuous Zn^2+^ conduction channels across the bulk.^[^ ^29^ ^]^ Together with CB, Figure 2b illustrates the coupled ion‐electron transport pathways and EAS region. In contrast, due to nonconductive molecular structure, (see Figure 2c), with electronegative fluorine atoms creating strong dipoles, PVDF network effectively impedes the flow of electrons and ions, thus limiting EAS to the cathode/electrolyte interface, see Figure 2d. It is, therefore, reasonable to expect that hydrogel cathode will outperform its PVDF counterpart in ZIBs.

**Figure 3:**
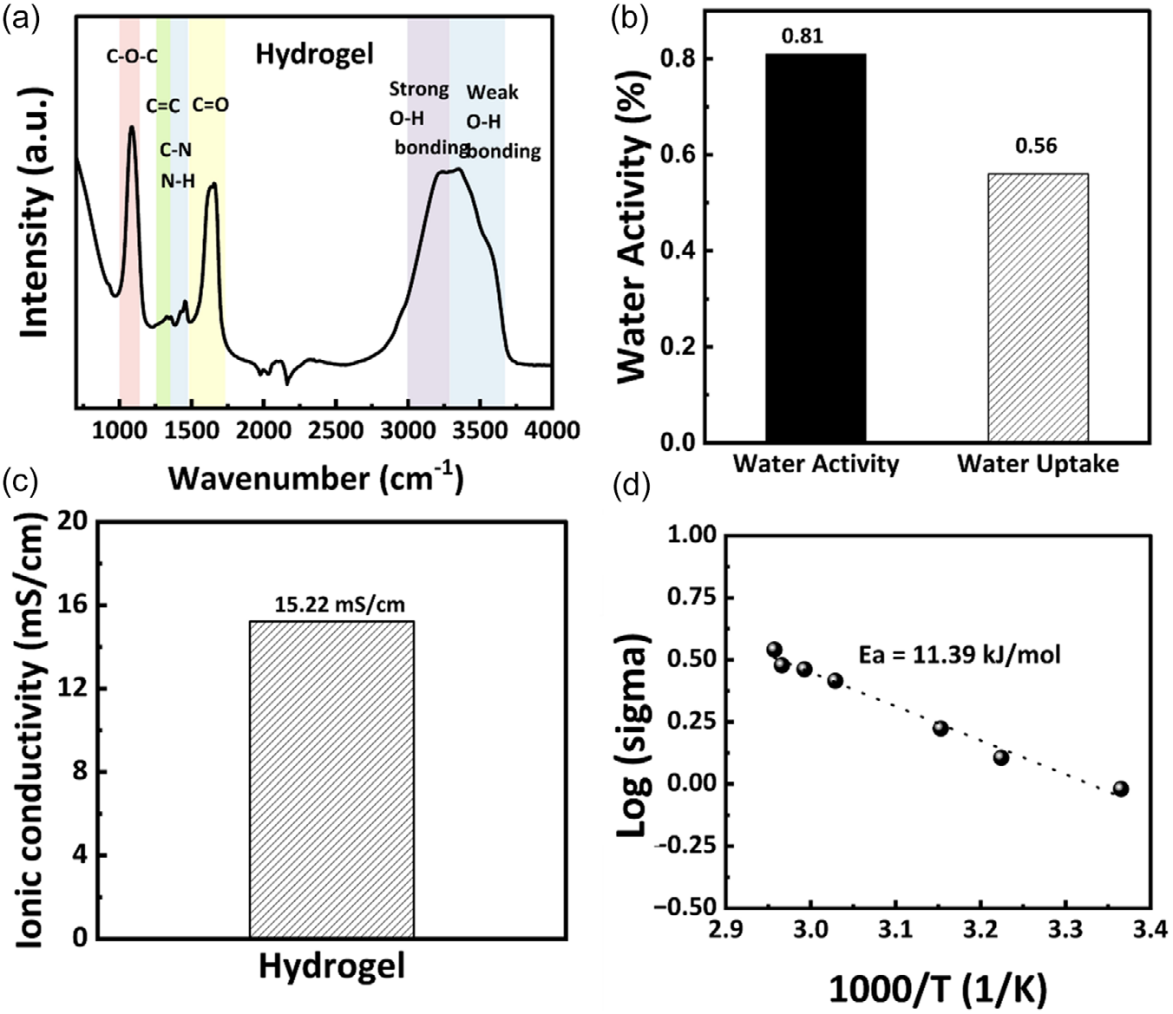
a) FTIR spectra of the hydrogel; b) water uptake and water activity of the hydrogel containing Zn(ClO_4_)_2_; ionic conductivity of the hydrogel containing Zn(ClO_4_)_2_; c) at room temperature; and d) as a function of temperature from 22 to 60 °C.

The synthesis process developed in this study (see Experimental Section) produced a composite hydrogel cathode membrane consisting of NVOH as the active material, a Zn^2+^‐conducting hydrogel backbone, and CB as the electron‐conducting phase. The functional groups within the hydrogel were confirmed by FTIR spectroscopy (**Figure** **3**a). A broad band at 3000–3500 cm^−1^ corresponds to the O—H and N—H stretching vibrations from –OH and –NH_2_ groups in CMCS and AM. The peak at 1650–1700 cm^−1^ is assigned to C=O stretching of carboxyl groups, while the features at 1400–1500 cm^−1^ are associated with N—H bending or C—N stretching.

The hydrogel exhibited a high water uptake of 56 wt% (Figure 3b), with a corresponding water activity (*a*_w_) of 0.81, indicative of a substantial proportion of free water, which is essential for efficient Zn^2^^+^ transport. Ionic conductivity measurements (Figure 3c,d) revealed a room‐temperature conductivity of 15.22 mS cm^−1^ (see EIS spectrum in Figure S1, Supporting Information) and an activation energy of 11.39 kJ mol^−1^ across 22–60 °C, underscoring the facile Zn‐ion transport enabled by the hydrogel matrix.

The XRD pattern of the as‐prepared NVOH active material (Figure S2, Supporting Information) confirms a single‐phase composition. Additionally, scanning electron microscopy imaging and energy‐dispersive X‐ray spectroscopy mapping (Figure S3a,b, Supporting Information) demonstrate uniform dispersion of CB and NVOH within the hydrogel matrix, further supporting the formation of a homogeneous, well‐integrated electrode microstructure with abundant three‐phase containing EAS.

### 3.2 Electrochemical Performance of the Hydrogel Cathode

A major challenge for aqueous ZIBs is the poor capacity stability, particularly at low current densities (≤1 A g^−1^). Low‐rate cycling is critical for long‐duration, high‐capacity energy storage applications; thus, improving the stability of ZIBs under these conditions is of practical importance.

**Figure 4:**
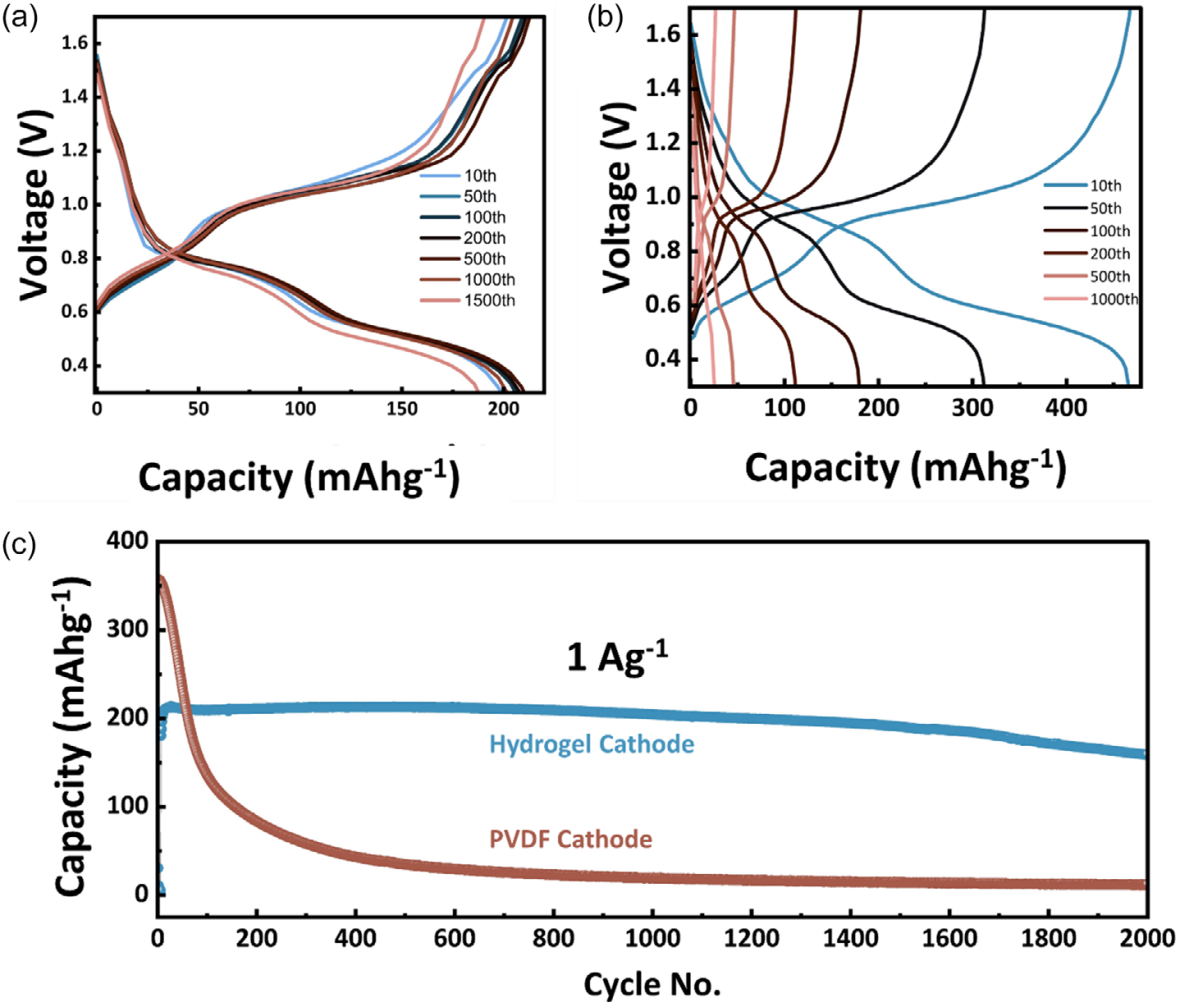
GCD profiles at 1.0 A g^−1^ of a) hydrogel cathode; b) PVDF cathode cycled at 1.0 A g^−1^; and c) cycling stability comparison. Electrolyte: 2 m Zn(ClO_4_)_2_/glass fiber separator. Anode: Zn metal.

**Figure** **4**a,b compare the GCD profiles of hydrogel‐ and PVDF‐based NVOH cathodes at 1.0 A g^−1^. The hydrogel cathode delivers a lower initial capacity but exhibits far superior stability, retaining 77% of its capacity after 2000 cycles, whereas the PVDF cathode loses nearly all capacity within 1000 cycles, see Figure 4c. This performance is also superior to previously reported PVDF‐based NVOH cathodes, which exhibited, for example, 28% capacity loss after 100 cycles at 1.0 A g^−1^^[^ ^30^ ^]^ and 60% loss after 1000 cycles at 2.0 A g^−1^.^[^ ^31^ ^]^

**Figure 5:**
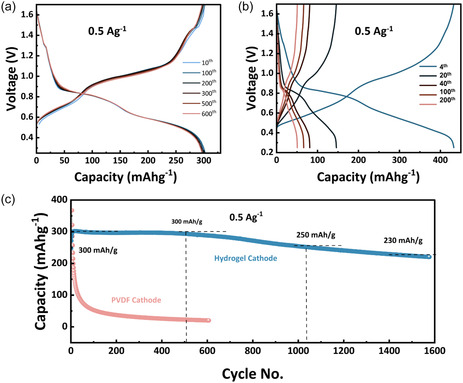
GCD profiles at 0.5 A g^−1^ of a) hydrogel cathode; b) PVDF cathode cycled at 0.5 A g^−1^; and c) cycling stability comparison. Electrolyte: 2 m Zn(ClO_4_)_2_/glass fiber separator. Anode: Zn metal.

The cycle stability at 0.5 A g^−1^ between the two cathodes is further compared in **Figure** **5**. The PVDF cathode delivers a higher initial capacity (>400 mAh g^−1^) but undergoes rapid decay, losing most of its capacity within only a few tens of cycles, underscoring its poor durability. In contrast, the hydrogel cathode exhibits a slightly lower initial capacity (≈300 mAh g^−1^) yet maintains outstanding long‐term stability—showing virtually no capacity loss after 600 cycles and retaining ≈230 mAh g^−1^ even after 1600 cycles. The stable voltage‐capacity profiles of the hydrogel electrode (Figure 5a) compared with the sharp degradation of the PVDF electrode (Figure 5b) clearly demonstrate that the hydrogel binder suppresses dissolution and sustains electrochemical activity over extended cycling. At the same current density of 0.5 A g^−1^, the hydrogel cathode preserves nearly 100% of its capacity after 600 cycles and ≈75% after 1400 cycles, whereas PVDF‐based cathodes with the same NVOH material typically lose ≈33% of their capacity after only 100 cycles at a lower rate of 0.2 A g^−1^.^[^ ^32^ ^]^

**Figure 6:**
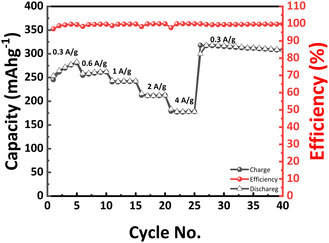
Capacity and Coulombic efficiency of the hydrogel cathode at different rates.

Rate performance is shown in **Figure** **6**, where it further highlights the robustness of the hydrogel cathode design. The specific capacities remain relatively insensitive to current density, delivering 271, 261, 241, 210, and 177 mAh g^−1^ at 0.3, 0.6, 1.0, 2.0, and 4.0 A g^−1^, respectively. Upon returning to 0.3 A g^−1^, the capacity increases from the original 271 to 320 mAh g^−1^, demonstrating both excellent rate capability and an activation effect during cycling.

The exceptional stability of the hydrogel cathode can be attributed to its abundant EAS. As illustrated in Figure 2b, the Zn‐ion‐conducting hydrogel matrix embeds both the active material NVOH and CB, effectively converting the bulk electrode into a continuous EAS network. In contrast, conventional PVDF‐based electrodes confine EAS to the electrolyte/cathode interface as illustrated in Figure 2d. The EIS spectra (Figure S4, Supporting Information) support this interpretation, showing significantly lower electrode resistance and better stability for the hydrogel cathode than PVDF‐based electrodes. Furthermore, analysis of the kinetic analysis in (Figure S5, Supporting Information) indicates that the hydrogel cathode exhibits a significant capacitance component, signaling high‐rate capability.

The ability of the hydrogel matrix to suppress dissolution of the active material, a major degradation mechanism in ZIB cathodes, is further verified by a soaking experiment. In this experiment, both hydrogel‐ and PVDF‐based cathodes were immersed in 2 m Zn(ClO_4_)_2_ electrolyte for three days. Figure S6, Supporting Information) shows a clear color change to yellow in the electrolyte containing the PVDF cathode, consistent with previous observations.^[^ ^33^ ^]^ This simple test confirms that the hydrogel matrix effectively protects active material by separating it from direct water attack. This suppression, coupled with the high density of EAS, accounts for the prolonged cycling stability observed. By contrast, in PVDF‐based cathodes, interfacial dissolution progressively reduces the number of active sites, leading to rapid capacity decay. Comparisons in Figure S4 and Table S2, Supporting Information, of charge‐transfer and ohmic resistances between the two cathodes further support the above conclusion.

**Figure 7:**
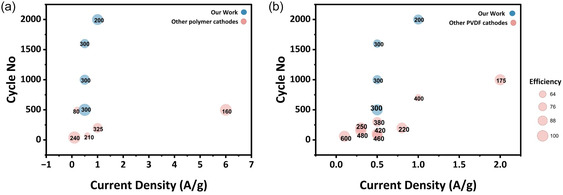
Comparison of hydrogel cathode with previous polymer cathodes. The size of the circles represents the efficiency after cycling, while the numbers inside the circles indicate the initial capacity (mAh g^−1^). Blue circles correspond to the results of our hydrogel cathode, and dark pink circles represent previous works with different active materials. a) Comparison with other polymer‐binder‐based cathodes. b) Comparison with PVDF‐binder‐based cathodes (ref. [12, 13, 18, 19, 25, 26, 28, 34, 35, 36, 37, 38, 39, 40]).

Lastly, the electrochemical performance of battery with hydrogel cathode is compared in **Figure** **7** with previously reported polymer‐ and PVDF‐binder cathodes. In these comparisons, the blue circles (our work) show markedly higher cycle numbers and stronger capacity retention compared to the pink circles (other reported works). The size of the circles represents efficiency after cycling, while the numbers inside the figures denote the initial capacity (mAh g^−1^). Importantly, regardless of which active material was used in previous reports, the binder appears to play a decisive role in determining long‐term stability. This underscores that the hydrogel binder, rather than the specific active material, is the dominant factor in enabling durable cycling.

## 4 Conclusion

In summary, we have demonstrated a new hydrogel‐based cathode that delivers markedly superior low‐rate cycling stability compared with conventional PVDF‐based counterparts. The enhanced performance originates from the Zn^2+^ conducting hydrogel, which establishes a continuous network of EAS throughout the electrode bulk. This contrasts sharply with PVDF‐based cathodes, where EAS are confined to the electrode/electrolyte interface. The combination of high EAS density, moderated water activity and suppressed dissolution enables prolonged cycling stability at low current densities. These findings address a key degradation challenge in aqueous ZIBs and highlight hydrogel cathodes as a promising pathway toward durable, long‐duration energy storage.

## Conflict of Interest

The authors declare no conflict of interest.

## Supporting information

Supplementary Material

## Data Availability

The data that support the findings of this study are available from the corresponding author upon reasonable request.
