# Supplementary material for: A New Low‐Rate Stable Hydrogel Cathode for Aqueous Zn‐ion Batteries
Source: ChemSusChem. 2025 Oct 23;18(24):e202501942. doi: 10.1002/cssc.202501942 (PMC12703429; doi:10.1002/cssc.202501942)
Supplement: Supplementary file 1 — Supplementary Material [file CSSC-18-e202501942-s001.pdf]

Supporting Information

A New Low-rate Stable Hydrogel Cathode for Aqueous Zn-ion Batteries

Roya Rajabi, Shichen Sun, Jamil Khan, Morgan Stefik[#], Kevin Huang*

Department of Mechanical Engineering, University of South Carolina, Columbia, SC29201.

[#]Department of Chemistry and Biochemistry, University of South Carolina, Columbia, SC29201.

*Corresponding author: huang46@cec.sc.edu

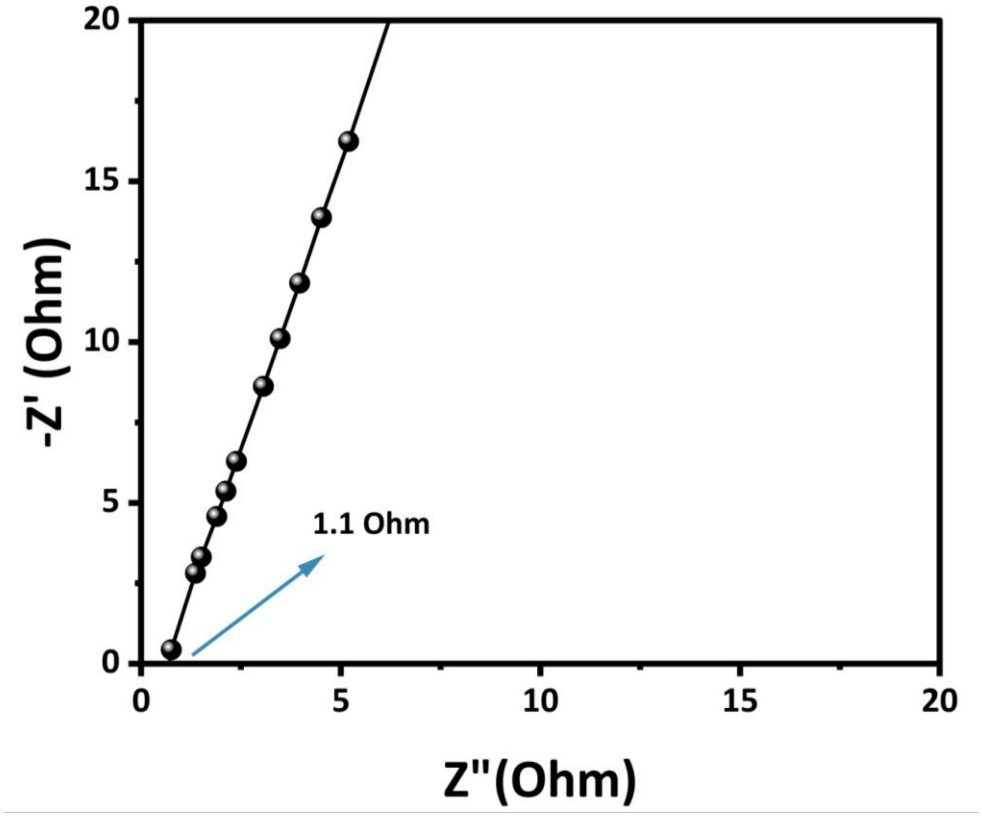

Figure S1. EIS spectrum of $Zn(ClO_4)_2$ doped hydrogel used in this study.

Table S1: Ohmic resistance of the hydrogel containing zinc salt at different temperatures.

**Temperature (K)**	**Ohmic Resistance (Ω)**
297	1.01
310	0.9
317	0.8
330	0.66
334	0.63
337	0.62
338	0.582

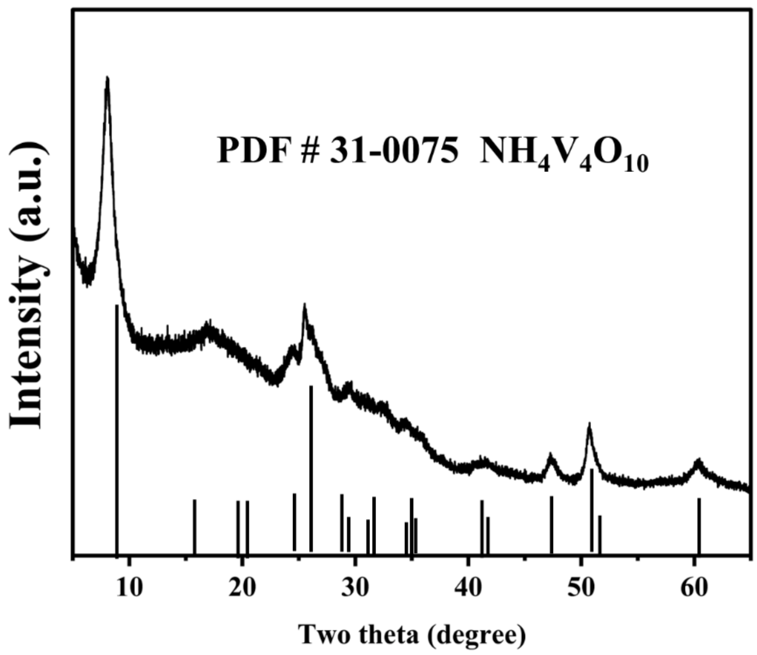

Figure S2. XRD pattern of NVOH synthesized.

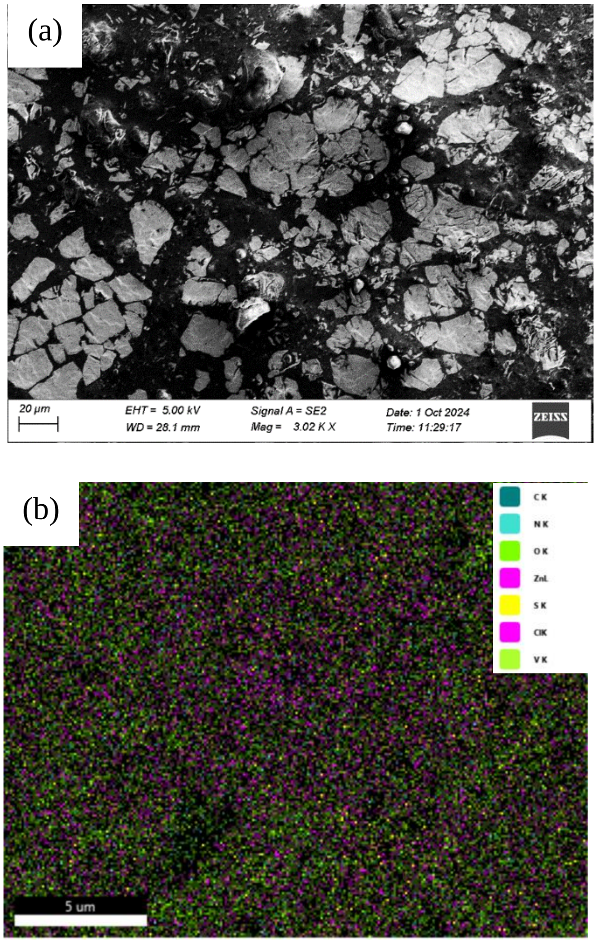

Figure S3. (a) SEM image and (b) EDX elemental mapping of hydrogel cathode before testing.

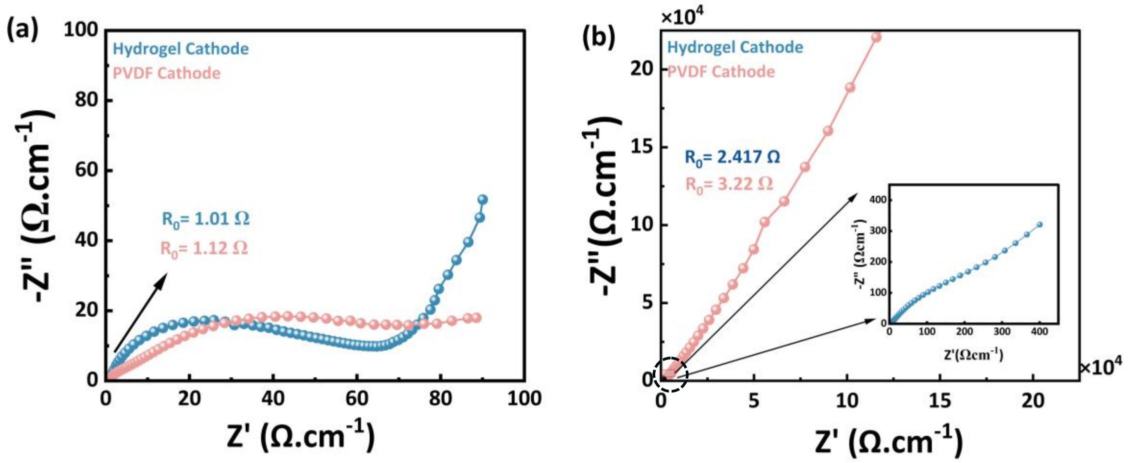

Figure S4. Comparison of impedance spectra of hydrogel and PVDF cathodes (a) before and (b) after cycling at 0.5 A·g^{-1} after 600 cycles for PVDF cathode and 1500 cycles for hydrogel cathode.

Table S2: Ohmic resistance of the hydrogel cathode and PVDF cathode before and after cycling at 0.5 Ag^{-1}

		**Ohmic Resistance (Ω)**
**Hydrogel Cathode**	Before Cycling	1.01
	After Cycling	1.12
**PVDF Cathode**	Before Cycling	2.417
	After Cycling	3.22

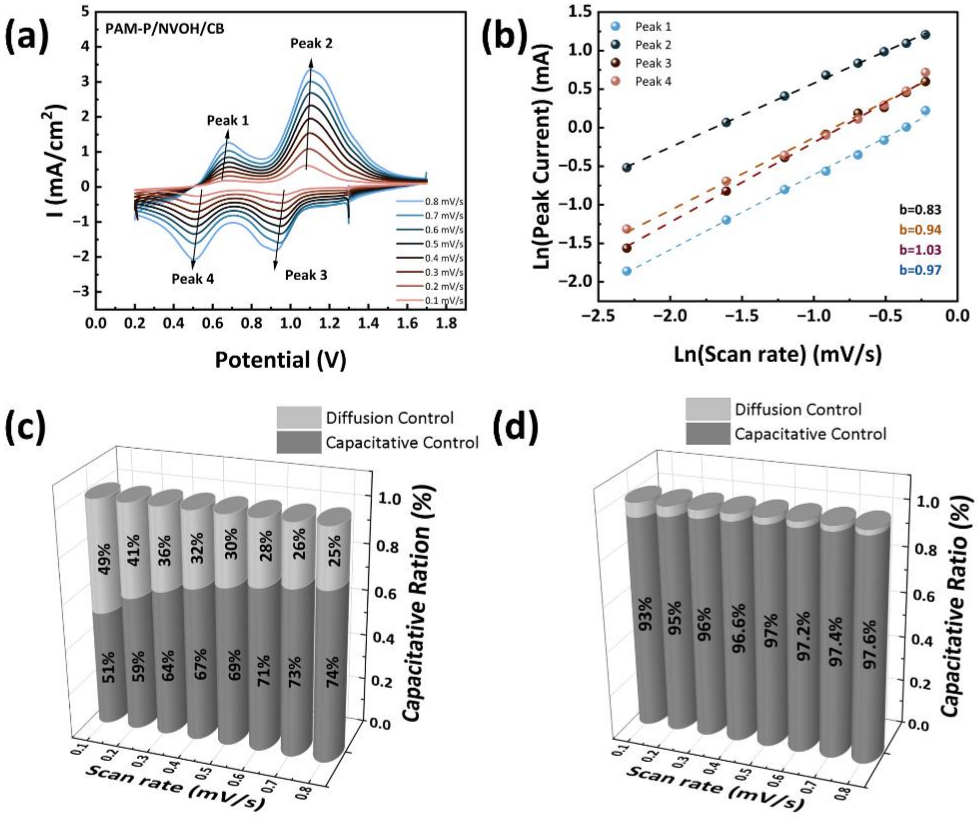

Figure S5: (a) CV curves of PAM-P/NVOH/CB at different scan rates; (b) Ln(i) versus Ln(scan rate) plot at different oxidation and reduction states; (c) capacitive contribution at oxidation state at various sweep rates; (d) capacitive contribution at reduction state at various sweep rates.

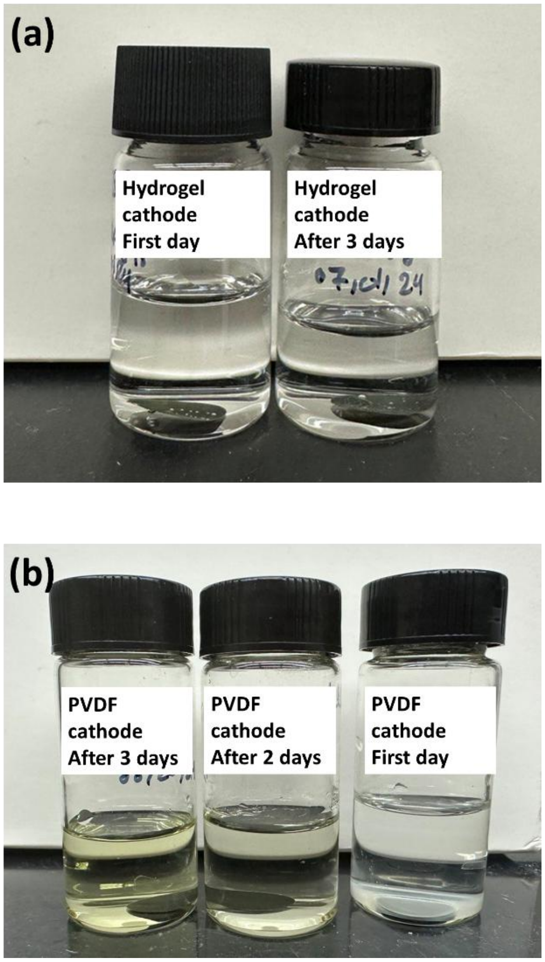

Figure S6: Cathode dissolution test: (a) hydrogel cathode, (b) PVDF cathode soaked in 2M $Zn(ClO_4)_2$ for 3 days.

Table S3: Comparison of the battery performance in this work compared to previous works with PVDF as binder and non-PVDF binder.

	Nom	Cathode	Rate (Ag^{-1})	Stability (efficiency)	Initial capacity (mAh/g)	Ref
PVDF Binder	1	$VO(OH)_2$	1	700 cycles (64%)	400	35
	2	HCLHATN	2	1000 cycles (100%)	175	36
	3	ZVO	0.3	150 cycles (100%)	480	37
	4	ZVO	0.5	300 cycles(86%)	380	38
	5	ZVO	0.5	100 cycles (100%)	420	38
	6	VO_2	0.1	60 cycles (100%)	~600	39
	7	CaMnO	0.3	200 cycles (99%)	250	40
	8	MnO-CNT-C_3N_4	0.8	200 cycles (96%)	220	12
	9	NVO-350	0.5	80 cycles (86%)	~460	13
Non PVDF Binder	10	CMC	0.2	500 cycles (50%)	~80	19
	11	SA+PTFE	0.1	40 cycles (100%)	240	29
	12	Zn–MnO_2/rGO	6	500 cycles (96%)	~160	25
	13	V_2O_5/CNT	1	2000 cycles (80%)	325	26
	14	HEC	0.6	70 cycles (54%)	210	30
	15	Our Work	0.5	500 cycles (100%)<br>1000 cycles (83%)<br>1600 cycles (76%)	300	This work
			1	2000 cycles (90%)	200	This work
